# Repeatability of Scotopic Sensitivity and Dark Adaptation Using a Medmont Dark-Adapted Chromatic Perimeter in Age-related Macular Degeneration

**DOI:** 10.1167/tvst.9.7.31

**Published:** 2020-06-25

**Authors:** Durin Uddin, Brett G. Jeffrey, Oliver Flynn, Wai Wong, Henry Wiley, Tiarnan Keenan, Emily Chew, Catherine Cukras

**Affiliations:** 1 National Eye Institute, NIH, Bethesda, Maryland, USA

**Keywords:** dark adaptation, repeatability, scotopic sensitivities, retina, age-related macular degeneration

## Abstract

**Purpose:**

Functional studies of rods in age-related macular degeneration using the Medmont Dark-Adapted Chromatic Perimeter (DACP) have demonstrated impairments in scotopic sensitivities and dark adaptation (DA). We investigated the intersession repeatability of scotopic sensitivity and DA parameters including the rod intercept time recorded from the Medmont DACP.

**Methods:**

Scotopic thresholds (14 test points) and DA using a 30% photobleach (eight test points) were measured on two separate days from participants 50 years of age or older with a range of age-related macular degeneration severity at loci superior and inferior to the fovea. Repeatability coefficients were calculated for prebleach scotopic sensitivity, and for DA parameters including rod intercept time.

**Results:**

Twelve participants (mean age, 79.7 ± 8.1 years) repeated Medmont DACP testing within 50 days. Repeatability coefficients for prebleach scotopic sensitivity to long wavelength (red, 625 nm) and short wavelength (cyan, 505 nm) were 5.9 dB and 7.2 dB, respectively. The DA curve-derived repeatability coefficients for cone threshold was 3.9 dB, final threshold 5.3 dB, with an R value of 0.075 decades/min, rod intercept time 7.6 minutes, and RITslope 0.54 min/degree.

**Conclusions:**

This study establishes repeatability coefficients for scotopic thresholds and multiple DA parameters obtained with the Medmont DACP in patients with age-related macular degeneration. These repeatability coefficients will serve as the basis for determining clinically meaningful change in rod function in future clinical trials.

**Translational Relevance:**

Measures of repeatability parameters of scotopic thresholds and DA are essential to the accurate interpretation of results in future studies and trials using these measures.

## Introduction

The visual symptoms first noted by individuals with age-related macular degeneration (AMD) often include difficulties adjusting to dim lighting and with driving at night.^1^^–^^7^ These symptoms typically occur in the absence of decreased visual acuity. Such changes in rod-mediated visual function are consistent with histologic evaluations of donor autopsy eyes showing early and preferential loss of rod over cone photoreceptors, a pattern that persists over the course of the disease.^8^^,^^9^ Psychophysical measures of rod-mediated function include scotopic sensitivity, which measures the maximum light sensitivity of the fully dark-adapted retina, and dark adaptation (DA), the rate of recovery of retinal sensitivity after exposure to an intense light.

Both scotopic sensitivity and DA worsen with advanced age but are most affected by the presence of disease (AMD); of the two parameters, DA is most significantly affected in AMD.^9^^–^^16^ Because slower DA reflects a disease-relevant functional change that parallels subjective visual symptoms under low luminance conditions,^7^^,^^17^ DA has been considered as a potential functional biomarker in intermediate AMD.^6^^,^^16^^,^^18^ In addition, the measurement of scotopic thresholds provides data on rod function that is complementary to DA measurements; investigating both parameters increases our understanding of changes in rod-mediated function in AMD.^19^

Historically, scotopic sensitivity has been measured using modified versions of commercial perimeters (e.g., the Tubingen perimeter,^20^ Humphrey perimeter,^10^^,^^21^^,^^22^ and Octopus perimeter^23^). In recent years, an increasing number of commercial instruments have become available, including the MP-1S (Nidek Technologies, Padova, Italy),^24^^,^^25^ the scotopic Macular Integrity Assessment (CenterVue, Padova, Italy),^26^^,^^27^ the MonCvONE (MetroVision, Perenchies, France), and the Medmont Dark-Adapted Chromatic Perimeter (DACP) (Medmont, Nunawading, Australia).^28^^,^^29^ There is, however, a relative paucity of test–retest variability data for scotopic measurements with these devices, with literature surveys revealing a single study each for AMD^28^ and retinitis pigmentosa^29^ with the Medmont DACP, a single study of retinitis pigmentosa with a modified Humphrey perimeter,^30^ and single studies of healthy volunteers, maculopathy and AMD patients with the scotopic Macular Integrity Assessment.^26^^,^^27^^,^^31^

Data on the test–retest variability of DA parameters are also scarce, with a single study of AMD patients with a prototype of the AdaptDx (MacuLogix, Harrisburg, PA)^16^ and one study of healthy volunteers with the Goldmann–Weekers adaptometer,^32^ which is no longer in production.

Scotopic thresholds and DA are altered along a steep gradient across retinal eccentricity from the fovea in AMD, with the largest deficits occurring nearest the fovea.^19^^,^^28^^,^^33^ By quantifying the different spatial variations in both scotopic threshold and DA with the Medmont DACP, we were able to further define rod function phenotypes in AMD. A detailed understanding of test–retest repeatability metrics for both scotopic sensitivity and DA, at different eccentricities from the fovea, is essential to the design of future studies and trials using these measures.

## Methods

### Study Population

Participants aged 50 years or older, both with and without AMD, were recruited from ongoing studies approved by the Institutional Review Board of the National Institutes of Health at the National Eye Institute, Bethesda, Maryland. The eligibility criteria for study eyes were (1) a best-corrected visual acuity of 20/63 or better with the ability to maintain foveal fixation, and (2) pupils achieving dilation to diameter 6.3 mm or greater, to ensure the necessary bleach levels.^15^^,^^31^ At the participant level, the exclusion criteria were (1) advanced AMD in both eyes, (2) any other active ocular or macular disease (e.g., glaucoma or diabetic retinopathy), (3) cataract surgery within 3 months before enrollment, (4) a history of vitamin A deficiency, (5) high oral intake of vitamin A palmitate supplement (≥8000 international units per day), and (6) active or history of liver disease.

This study adhered to the tenets set forth by the Declaration of Helsinki and was Health Insurance Portability and Accountability Act compliant. Written informed consent was obtained from all participants.

### Ophthalmic Examination and Imaging

Testing occurred between June 1, 2017, and November 30, 2018. All participants underwent a complete ophthalmoscopic examination and retinal imaging including color fundus photography, fundus autofluorescence, infrared reflectance, and spectral-domain optical coherence tomography imaging (as described in detail elsewhere^16^). Participants contributed only one study eye to the analysis. Study eyes were evaluated for the presence of subretinal drusenoid deposits (SDD), using all four image modalities, according to previously defined criteria and these eyes were placed in a separate group.^16^ Eyes without SDD were categorized into two groups: intermediate AMD (maximum soft drusen diameter of >125 µm) or controls (no soft drusen with a diameter of >125 µm in either eye). Fellow eyes could have any level of AMD, including advanced AMD.

### Medmont DACP Scotopic Threshold Testing and Analysis

Scotopic sensitivity was measured with the Medmont DACP in the study eye using a protocol described in detail previously.^19^ In brief, following pupil dilation, participants waited in the dark for 30 minutes. Scotopic sensitivities were then measured at 14 retinal locations: 2°, 4°, 6°, 8°, 10°, 12°, and 18° superior and inferior to the fovea along the vertical meridian. Scotopic sensitivities were first recorded to a red stimulus (dominant wavelength of 625 nm) and then to a cyan stimulus (dominant wavelength of 505 nm). The maximum photopic luminance for the cyan stimuli was 12.58 cd/m^2^ with a test range of 0 to 75 dB attenuation. For the red stimuli, the maximum luminance was 4.64 cd/m^2^ with a range of 0 to 50 dB. The principle of two color dark-adapted perimetry (i.e., the difference between red and cyan sensitivities) can be used to discern whether the responses at each locus are mediated by rods, cones, or a mixture of rods and cones.^27^^,^^34^^–^^37^

### Medmont DACP DA Testing and Analysis

DA was measured and analyzed as described in detail previously.^19^ In brief, study eyes were exposed to a full-field background bleach of 347 scotopic candelas per meter squared (for 5 minutes, equivalent to a 30% rhodopsin bleach as outlined in the Appendix of Flynn et al.^19^). Following the bleach exposure, sensitivities to the cyan stimulus were measured at eight retinal locations along the vertical meridian (4°, 6°, 8°, and 12° superior and inferior to the fovea) for 30 to 45 minutes.

**Figure 1.**
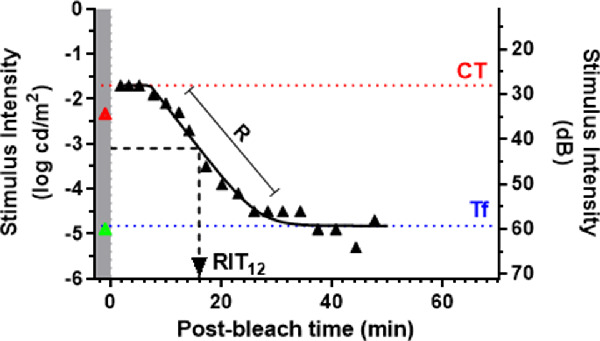
Medmont DA curve at 12° showing the curve-derived parameters measured. The *gray region* with the *green* and *red triangles* illustrates the measurement of scotopic thresholds at 505 nm and 625 nm respectively before delivery of bleach. The RIT (minutes) is the time to detect a stimulus of −3.1 photopic cd/m^2^. The Tf (log cd/m^2^ or dB) is final rod-mediated threshold. CT (log cd/m^2^ or dB) is the cone threshold. R (decades/min) is the rate of rod decay in the S2 portion of the DA curve.

Figure 1 illustrates the derived parameters used to quantify DA. The rod intercept time (RIT) is the time (in minutes) taken to detect a criterion stimulus of –3.1 log photopic cd/m^2^ (44 dB attenuation on the Medmont device); this has been used widely to assess DA in AMD.^3^^,^^16^^,^^19^^,^^38^^,^^39^ Other derived parameters include: cone sensitivity (dB), measured from the first plateau prior to the rod-cone break; final asymptotic sensitivity (Tf) (dB), and R (decades/min), the rate of rod decay representing the slope of the rod-mediated second component of DA described by Lamb.^40^ We also calculated RITslope, which is the linear fit of RIT as a function of decreasing retinal eccentricity.^19^

The following criteria were established to ensure sufficient data were available from a test to reliably derive a value for each parameter:

1. Cone sensitivities were only measured from DA tests in which at least three points clearly established a plateau prior to the rod–cone break.
2. Tf values greater than the range that could be measured (75 dB) were excluded. In such cases, Tf was constrained to the prebleach scotopic threshold.
3. R was only derived if there was a sequential reduction in threshold for at least three values in the linear portion of the DA test after the rod–cone break.
4. RIT values were excluded if the first scotopic sensitivity measured after the bleach exceeded the criterion sensitivity of 44 dB; RIT by definition could not be derived if scotopic sensitivity (before bleach or after bleach) never reached the 44 dB criterion.
5. The RITslope was only calculated in a hemisphere (superior or inferior) when RIT could be derived for at least three retinal eccentricities.

### Statistical Analysis

Repeatability coefficients (RCs) were calculated as two times the standard deviation of the difference between two measurements (v_2_ and v_1_) ^41^^–^^43^:

(1) $$\mathrm{RC}=1.96\times \sqrt{\frac{\sum {\left(v_{2}-v_{1}\right)}^{2}}{n}}$$

Where *n* is number of participants. Equation 1 is applicable for repeatability measurements because the mean difference between two measures on the same participant should be zero. RCs were calculated for the Medmont DACP prebleach scotopic sensitivities, cone sensitivities, final sensitivities, and R values.

## Results

### Participant Demographics

Twelve participants (age range, 63–90 years; mean, 79.7 ± 8.1 years) completed Medmont DACP testing at two separate sessions within 50 days. The study population was 100% Caucasian, and 41.7% of participants were female. The 12 participants had a range of AMD severity: four participants had intermediate AMD with large drusen in the study eye, without the presence of SDD; five participants had SDD in the study eye; and three participants had no large drusen, SDD, or advanced AMD in the study eye and served as controls.

### Scotopic Sensitivity

A two-way analysis of variance was used to examine whether scotopic sensitivity varied with retinal eccentricity and/or between the two visits. There was no significant difference in the scotopic sensitivity for the red stimuli from the inferior retina, nor for the cyan stimuli from either hemisphere. In comparing the values obtained from the two visits, there was a small but significant decrease in scotopic sensitivity for red stimuli in the superior retina in visit two compared with visit one (visit one, mean of 32.6 ± 4.6 dB; visit 2, mean of 31.4 ± 4.6 dB; *P* = 0.001). Together, these results suggest there was no learning effect across the two visits on scotopic sensitivity measurements.

Table 1 shows the RCs for both the cyan and red stimuli and for the difference in sensitivity between these two stimuli (cyan–red), at each retinal eccentricity. RCs did not correlate with eccentricity, although the highest RCs for each condition were observed at 2° from the fovea.

**Table 1.** Repeatability Coefficients for Scotopic Sensitivity

Eccentricity (°)	Cyan RC (dB)	Red RC (dB)	Cyan-Red RC (dB)
18	7.5	5.7	8.4
12	6.6	6.6	8.5
10	7.8	4.9	8.7
8	6.2	6.0	8.2
6	7.3	5.6	8.8
4	5.8	4.1	6.4
2	8.7	7.8	10.4
All eccentricities combined	7.2	5.9	8.5

RC: repeatability coefficient.

**Figure 2.**
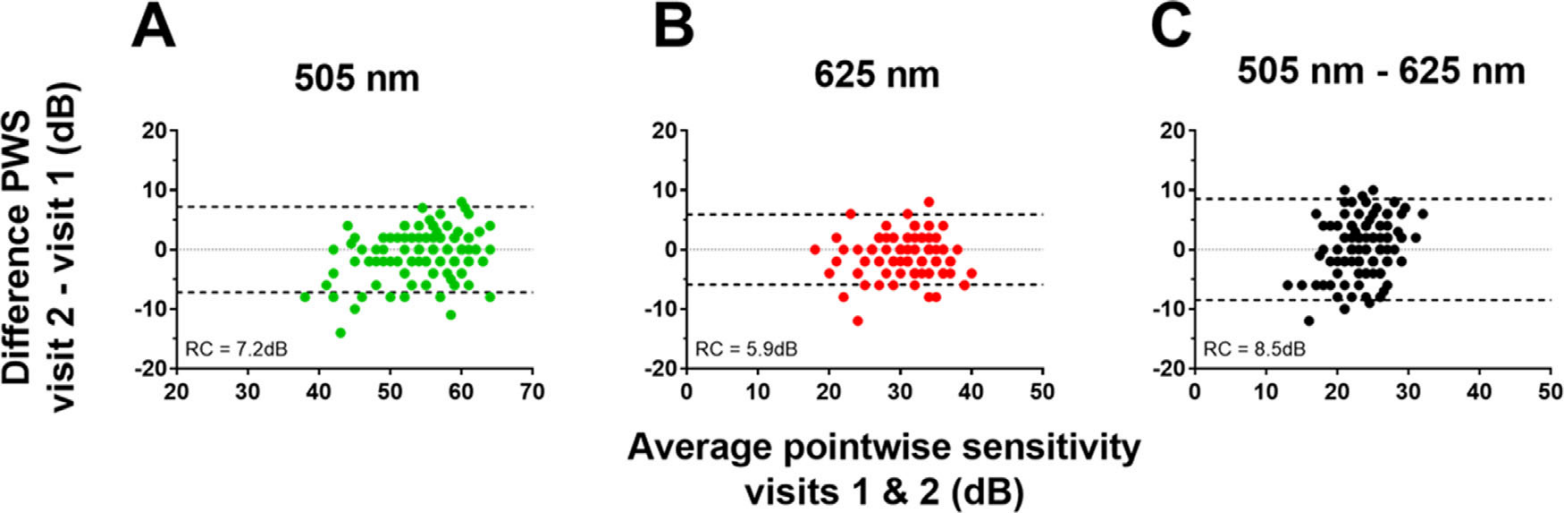
Assessment of scotopic threshold repeatability. Bland-Altman plots of scotopic thresholds for the 505 nm (A) and 625 nm (B) stimuli. A Bland–Altman plot was also calculated for the difference in thresholds (505–625 nm) for each point tested (C). The thick dashed lines are set at the values of the repeatability coefficients calculated for each stimulus condition.

We combined repeatability data across all eccentricities for each stimulus to calculate an overall RC for that stimulus condition (Table 1). The RC was 5.9 dB for the red stimulus, 7.2 dB for the cyan stimulus, and greatest at 8.5dB for the cyan–red difference. Figure 2 demonstrates these data graphically.

The RC calculations shown in Table 1 represent a statistical approach to the study of variability. We also took a more heuristic approach to determine the proportion of test points producing a variability of 4 dB or less: this occurred in 82% of the points with the cyan stimuli, 91% with the red stimuli, and 73% with the cyan–red difference.

### Dark Adaptation

Table 2 summarizes the percentage of points for which DA parameters could be derived using the criteria outlined in the Methods section. Study eyes with fast DA (healthy volunteers and some participants with AMD) did not demonstrate a definite cone plateau (50% of loci from healthy volunteers and 39% of loci from AMD) (Table 2). In contrast, a sustained cone plateau was observed in most study eyes with SDD, and a cone threshold could be measured at 88% of loci from these eyes. There was little or no evidence of DA at 25% of loci from eyes with SDD and, therefore, no RIT could be derived in these eyes. The RITslope is calculated from multiple RIT values across the hemisphere. Because of the reduced number of loci for which RIT could be calculated for study eyes with SDD, RITslope could only be derived for 70% of hemispheres of these eyes.

**Table 2.** Percentage of Loci for Which Parameters Could Be Derived From DA Curves

	Control (*N* = 48) Loci,	AMD (*N* = 64) Loci,	SDD (*N* = 80) Loci,	All (*N* = 192) Loci,
DA	*N* (%) of Loci With	*N* (%) of Loci With	*N* (%) of loci with	*N* (%) of loci with
Parameter	Derived Points	Derived Points	Derived Points	Derived Points
CS	24 (50)	39 (61)	70 (88)	133 (69)
Tf	48 (100)	62 (97)	73 (81)	183 (95)
RIT	46 (96)	64 (100)	60 (75)	170 (89)
R	48(100)	64 (100)	70 (88)	180 (94)
RITslope^*^	12/12(100)	16/16 (100)	14/20 (70)	42/48 (88)

CS, cone sensitivity; R: rod recovery constant; RITslope: RIT slope.

* The RITslope is based on results from hemispheres (superior/inferior) not loci.

**Figure 3.**
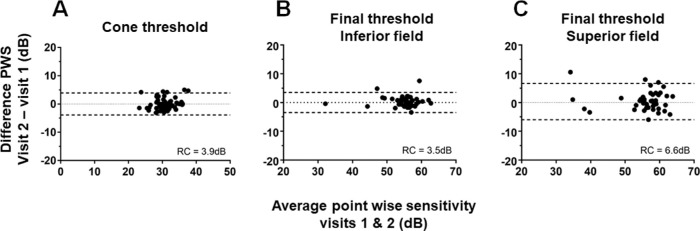
Assessment of calculated post-bleach threshold repeatability. Bland-Altman plots for (A) cone sensitivity and final sensitivity (B) and (C). Inferior field (B) and superior field (C) repeatability parameters are reported separately due to differences in the RC for the two hemifields.

The RCs for scotopic cone sensitivity did not vary with retinal eccentricity (Supplementary Table S1). Combining repeatability data across all eccentricities produced an overall RC of 3.9 dB for cone sensitivity (Table 3). The low variability in scotopic cone sensitivity measures is illustrated in Figure 3A.

**Table 3.** Repeatability Coefficients of DA Parameters

DA Parameter	RC
Cone threshold	3.9 dB
FT: superior retina	6.6 dB
FT: inferior retina	3.5 dB
RIT	7.6 min
R	0.075 dec/min
RITslope	0.54 min/deg

FT: final threshold; RIT: Rod intercept time; R: rod recovery constant; RITslope: RIT slope.

There was a strong hemisphere difference for final asymptotic sensitivity with considerably lower variability in the inferior retina (RC = 3.5dB) compared with the superior retina (RC = 6.6 dB) (Table 3 and Figures 3B and C)). However, final asymptotic sensitivity did not vary significantly with retinal eccentricity within a hemisphere (Supplementary Table S2).

**Figure 4.**
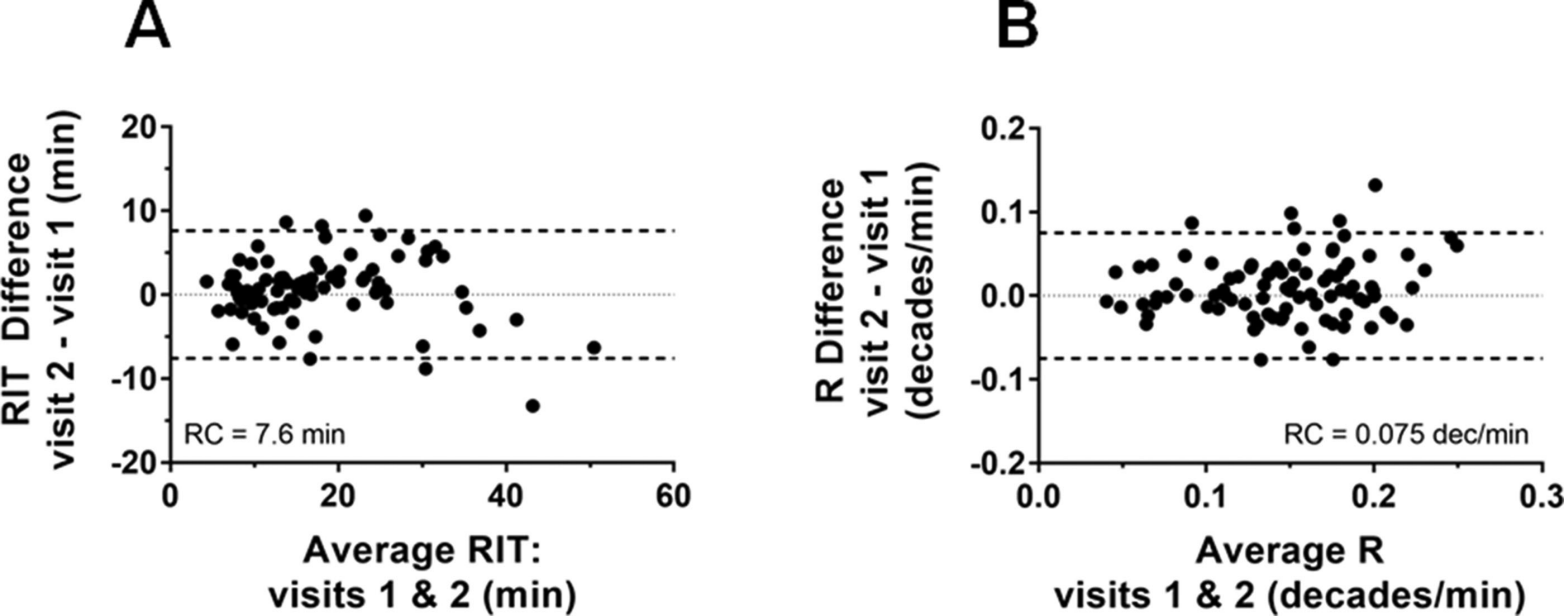
Assessment of repeatability of kinetic parameters of DA: Bland-Altman plots for (A) RIT (rod intercept time) and (B) R (rod decay) measures.

Figure 4 shows the Bland–Altman plots for RIT and R combined across all eccentricities. The RC for RIT was 7.6 minutes and RC for R was 0.075 dec/min (Table 3). There was no significant effect of eccentricity and/or hemisphere on RCs of either RIT or R (Supplementary Tables S3 and S4, respectively).

**Figure 5.**
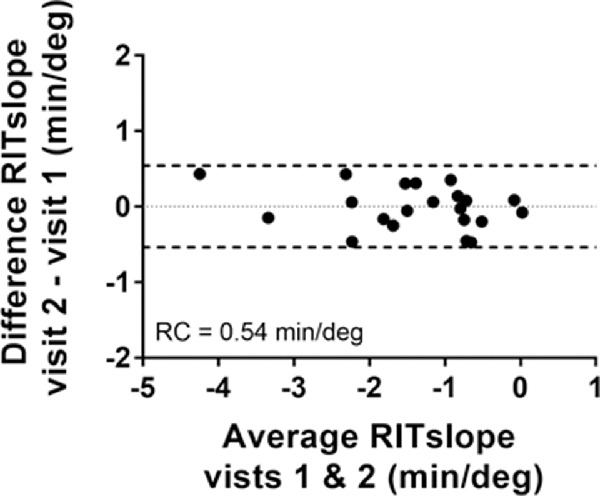
Assessment of the repeatability of the RITslope, a measure of spatial changes in the RIT across a hemisphere of the macula using a Bland–Altman plot.

The RITslope represents the gradient of RIT along the eccentricities tested. We derived the RITslope for each hemisphere of each study eye across the two visits and find the RC for RITslope to be 0.54 min/deg (Table 3 and Figure 5). These similarly did not appear to have significant effects of eccentricity or hemispheres on RCs (Supplementary Tables S5 and S6).

## Discussion

In this study, we established the repeatability ranges for scotopic sensitivities and multiple DA parameters obtained using the Medmont DACP. Both measures have been used by multiple research groups to investigate rod function in AMD and other retinal diseases.^16^^,^^19^^,^^28^^,^^29^^,^^33^ Establishing the variance parameters between testing trials is an essential part of benchmarking, that is, understanding what difference from baseline would represent a significant change in real-world settings. Understanding the intervisit repeatability of these measures will help facilitate the adoption of these measures in clinical trials, the interpretation between groups, and the evaluation of the values as outcome measures.

Our study found that scotopic threshold sensitivities in response to the red stimulus were more repeatable than those in response to the cyan stimulus which is similar to the findings of Tan et al.,^28^ using the Medmont DACP, and Pfau et al.,^26^ using the scotopic Macular Integrity Assessment. Our red stimulus scotopic threshold RC of 5.9 dB was comparable to the intersession RC values reported by Tan et al.^28^ (control participants [6.2 dB] and individuals with AMD [8.4 dB]) and larger than the red stimulus RC in the two studies with the modified MAIA.^26^^,^^44^ Our cyan stimulus scotopic threshold RC of 7.2 dB was also comparable to previous reports of repeatability parameters in the study by Tan et al. (control participants [RC 8.2 dB] and participants with AMD [RC 11.7 dB]).^28^ Similarly, a recent paper by Bennett et al.,^29^ studying intrasession and intersession repeatability using a cyan stimulus with the Medmont DACP in normal patients and patients with inherited retinal degenerations, reported an intersession central point-wise sensitivity RC of 6.8 dB in control eyes and 8.0 dB in eyes with inherited retinal degeneration. Our RC for the cyan–red difference was larger than the RC of either stimulus alone and likely represents the combination of variabilities. We summarize the results of these previous publications and instruments and our own in Table 4.

**Table 4.** Summary of Repeatability Studies of Scotopic Sensitivity Testing

			Age (y),			Test		Scotopic
			Mean ± SD			Points		CR505 nm/
Study	Population	*N*	[Range]	Visual Acuity	Perimeter	(n/radius [^o^])	Mesopic	625 nm
Current Study	Controls/iAMD/SDD	12	80 [63–90]	>20/63	Medmont DACP	14/18		7.2/5.9
Tan (2018)^28^	iAMD Controls	7 13	71 [62–82] 65 [59–79]	>20/60	Medmont DACP	28/24		11.7/8.4 8.2/6.2
Bennett (2019)^29^	IRD Controls	14 10	33 ± 15 40 ± 15	20/16–20/500	Medmont DACP	103 (144 x 72 grid)		9.8/− 6.8/−
Cideciyan (2018)^30^	XLRP	26 eyes	30 [19–42]	NR	Custom HVF	102 (168 x 84 grid)		9.6/−
Pfau (2017)^26^^,^^36^	iAMD controls	23 24	67 [50–85] 61 [50–71]	>20/50	S-MAIA	33/7	4.5 4.0	−/4.5 −/4.6
Pfau (2017)^27^	Macular disease	52	62 [19–90]	NR	S-MAIA	61/9	5.8	4.7/4.8
Pfau (2017)^26^	HV	30	NR	>20/20	S-MAIA	49/7	4.8	5.3/4.1

HV, healthy volunteer; HVF, Humphrey Visual Field; iAMD, intermediate AMD; IRD, inherited retinal degeneration; SD, standard deviation; S-MAIA, scotopic Macular Integrity Assessment; XLRP, X-linked retinitis pigmentosa.

We also compared Tf with prebleach scotopic sensitivities in response to the cyan stimulus. Similar to the findings in Flynn et al.,^19^ we found that 95% of the differences between the Tf and the scotopic threshold fell between –7.6 and 5.8 dB (Supplementary Fig. 1). Because we demonstrated that the Tf closely resembles the prebleach scotopic threshold, we were justified in using scotopic sensitivities to approximate the Tf when manual curve fitting was required. Our findings also suggest that cone threshold is more repeatable than final threshold (RC of 3.9 dB vs 5.3 dB).

The repeatability of DA curve-derived parameters as measured using a bleach in combination with the Medmont DACP has not, to our knowledge, been previously studied. An investigation of the intersession repeatability of a single measure, RIT, as measured using a prototype of the AdaptDx device, was performed in 87 patients with a range of AMD severity and reported a RC of 4.4 minutes.^16^ Although it is difficult to know the sources driving the differences in the RIT (RC on the prototype AdaptRx device [4.4 minutes] vs. the RIT RC in this study using the Medmont DACP [7.6 minutes]), some contributors could be the smaller sample size in this study and the greater number of eccentricities tested, leading to fewer points of the adaptation curves contributing to the mathematical fits, potentially leading to greater variability.

To our knowledge, the repeatability of other measures of DA, such as R (the rate of rod decay), have not been previously investigated. Similarly, the RITslope, which provides a measure of DA across the macula rather than a single test locus, has not been investigated for its repeatability. Because we previously reported that participants from increased severity AMD groups had points where the RIT (and even R) could not be calculated, the measure of RITslope has the potential to be a useful parameter.^19^ The RC of 0.54 min/deg found for RITslope is consistent with RIT repeat values that vary by up to 7 minutes.

Limitations of this study include the small sample size for Medmont-derived repeatability parameters and having too few participants to analyze subgroups of AMD, SDD, and controls separately. We have therefore reported our data as the repeatability of an aggregate group consisting of varying AMD severity. Furthermore, we analyzed RC by eccentricity along the vertical meridian rather than by rings surrounding the fovea, so our smaller number of data points may have masked repeatability differences in scotopic sensitivities for central compared with peripheral eccentricities in the macula reported in Tan et al.^28^

Establishing guidelines for interpreting scotopic threshold and DA curve-derived data obtained from the Medmont is crucial for guiding researchers in the field of DA as use of this device becomes more common. Repeatability studies have the potential to inform researchers in choosing which parameters and instruments are appropriate for a given research question. Knowledge about repeatability is vital to the development of multicentered studies and allow for the accurate interpretation of results in future studies that make use of these instruments.

## Supplementary Material

Supplement 1

Supplement 2

Supplement 3

Supplement 4

Supplement 5

Supplement 6

Supplement 7
