# Supplementary material for: Repeatability of Scotopic Sensitivity and Dark Adaptation Using a Medmont Dark-Adapted Chromatic Perimeter in Age-related Macular Degeneration
Source: Transl Vis Sci Technol. 2020 Jun 25;9(7):31. doi: 10.1167/tvst.9.7.31 (PMC7414623; doi:10.1167/tvst.9.7.31)
Supplement: Supplement 2 [file tvst-9-7-31_s002.docx]

| **Eccentricity** | **Cone Threshold RC (dB)** | N | |
| --- | --- | --- | --- |
| 12 superior | 3.3 | 7 |  |
| 8 superior | 3.5 | 8 |  |
| 6 superior | 3.9 | 10 |  |
| 4 superior | 2.4 | 9 |  |
| 4 inferior | 4.3 | 8 |  |
| 6 inferior | 4.4 | 8 |  |
| 8 inferior | 4.3 | 7 |  |
| 12 inferior | 6.7 | 2 |  |

**Table S1 : Cone Threshold repeatability at each test point**
