# Supplementary material for: Repeatability of Scotopic Sensitivity and Dark Adaptation Using a Medmont Dark-Adapted Chromatic Perimeter in Age-related Macular Degeneration
Source: Transl Vis Sci Technol. 2020 Jun 25;9(7):31. doi: 10.1167/tvst.9.7.31 (PMC7414623; doi:10.1167/tvst.9.7.31)
Supplement: Supplement 3 [file tvst-9-7-31_s003.docx]

| **Eccentricity** | **Final threshold RC (dB)** | **N** |
| --- | --- | --- |
| 12 superior | 6.3 | 12 |
| 8 superior | 6.1 | 11 |
| 6 superior | 8.9 | 10 |
| 4 superior | 4.4 | 10 |
| 4 inferior | 5.7 | 11 |
| 6 inferior | 2.8 | 12 |
| 8 inferior | 2.7 | 11 |
| 12 inferior | 1.9 | 11 |

**Table S2: Final calculated threshold repeatability at each test point**
