# Supplementary material for: Repeatability of Scotopic Sensitivity and Dark Adaptation Using a Medmont Dark-Adapted Chromatic Perimeter in Age-related Macular Degeneration
Source: Transl Vis Sci Technol. 2020 Jun 25;9(7):31. doi: 10.1167/tvst.9.7.31 (PMC7414623; doi:10.1167/tvst.9.7.31)
Supplement: Supplement 7 [file tvst-9-7-31_s007.docx]

|  | **RIT slope RC** | **N** |
| --- | --- | --- |
| Superior | 0.498 | 10 |
| Inferior | 0.565 | 11 |

Table S6: RIT slope repeatability in each hemisphere
